# Effects of a High-Fat Diet on Spontaneous Metastasis of Lewis Lung Carcinoma in Plasminogen Activator Inhibitor-1 Deficient and Wild-Type Mice

**DOI:** 10.1371/journal.pone.0110869

**Published:** 2014-10-30

**Authors:** Lin Yan, Lana C. DeMars

**Affiliations:** Grand Forks Human Nutrition Research Center, United States Department of Agriculture, Agricultural Research Service, Grand Forks, North Dakota, United States of America; University of L’Aquila, Italy

## Abstract

This study investigated the effects of a high-fat diet on spontaneous metastasis of Lewis lung carcinoma (LLC) in plasminogen activator inhibitor-1 deficient (PAI-1^−/−^) and wild-type mice. The high-fat diet increased the number of pulmonary metastases by 60% (*p*<0.01), tumor cross-sectional area by 82% (*p*<0.05) and tumor volume by 130% (*p*<0.05) compared to the AIN93G diet. Deficiency in PAI-1 reduced the number of metastases by 35% (*p*<0.01) compared to wild-type mice. In mice fed the high-fat diet, PAI-1 deficiency reduced tumor cross-sectional area by 52% (*p*<0.05) and tumor volume by 61% (*p*<0.05) compared to their wild-type counterparts; however, PAI-1 deficiency affected neither area nor volume in mice fed the AIN93G diet. Adipose and plasma concentrations of PAI-1 were significantly higher in high-fat fed wild-type mice than in their AIN93G-fed counterparts. Adipose and plasma PAI-1 were not detectable in PAI-1^−/−^ mice regardless of the diet. Mice deficient in PAI-1 showed significantly greater plasma concentrations of monocyte chemotactic protein-1, tumor necrosis factor-α, leptin, vascular endothelial growth factor, tissue inhibitor of metalloproteinase-1 and insulin compared to wild-type mice, indicating a compensatory overproduction of inflammatory cytokines, angiogenic factors and insulin in the absence of PAI-1. We conclude that PAI-1 produced by the host, including that by adipose tissue, promotes high-fat enhanced metastasis of LLC.

## Introduction

Plasminogen activator inhibitor-1 (PAI-1), a serine protease inhibitor, is a major component of the urokinase plasminogen activation system. It is the principal inhibitor of urokinase-type plasminogen activator (uPA) that catalyzes the conversion of latent plasminogen to plasmin, which is responsible for proteolysis of extracellular matrix during cancer invasion and metastasis. Paradoxically, PAI-1 may participate in the pathophysiology of cancer progression, because elevated PAI-1 expression in primary tumors is associated with poor prognosis and substantially increased risk of recurrence [1], [2]. Laboratory studies have found that PAI-1 promotes tumor growth [3] and experimental metastasis [4] and that depletion of PAI-1 inhibits tumor growth [5], invasion and vascularization [6] and metastasis [7]. However, others have reported that PAI-1 did not affect tumor growth [8] and metastasis [8], [9].

Recurrent or metastatic cancer, the spread of malignant cells from a primary tumor to different sites of the same organ or to distant organs, remains the most devastating aspect of cancer. Its occurrence directly affects the prognosis and survival of cancer patients, presenting a great challenge not only for cancer patients but also for physicians and researchers. Metastasis is a multistep process, from dissemination of malignant cells from a primary tumor, to intravasation into blood circulatory system, arrest in a distant vascular bed, extravasation into the interstitium of a target organ and proliferation to form metastases in the target organ. Stimulation of any of these steps can be expected to enhance malignant spread, whereas inhibition of any can be expected to reduce metastasis.

Obesity is a leading risk factor for cancer, second only to smoking. Being obese at the time of diagnosis of primary cancer can be predictive of poor prognosis. For example, breast cancer patients who are obese are at a greater risk of recurrence [10] with a shorter disease-free interval than those with normal body weight [11], and obese or overweight prostate cancer patients are more likely to have recurrence after radical prostatectomy than those of normal weight [12], [13].

Adipose tissue has been considered an endocrine organ that produces adipokines contributing to obesity. The expression of the adipokine PAI-1 is elevated in obese subjects [14], [15], and plasma levels of PAI-1 are elevated in mice fed obesigenic, high-fat diets [16], [17]. We reported that a high-fat diet enhances the malignant spread of Lewis lung carcinoma (LLC) in mice and this enhancement is accompanied by increases in plasma concentrations of PAI-1 [18], [19]. We hypothesized that PAI-1 participates in the spread of LLC and that the pro-metastatic effect of a high-fat feeding involves the up-regulation of PAI-1. The present experiments were conducted to test that hypothesis in PAI-1 deficient mice using a spontaneous metastasis model.

## Materials and Methods

This study was approved by the Animal Care and Use Committee of the U.S. Department of Agriculture, Agricultural Research Service, Grand Forks Human Nutrition Research Center. The procedures followed the National Institute of Health guidelines for the care and use of laboratory animals [20].

### Animals and diets

Four to five-week-old male PAI-1 deficient mice (PAI-1^−/−^, B6.129S2-*Serpine1^tm1Mlg^*/J) with a C57BL/6J background and C57BL/6J wild-type mice were purchased from The Jackson Laboratory (Bar Harbor, ME). The AIN93G diet [21] and AIN93G diet modified to include 45% of energy from dietary fat (hereafter referred to as the high-fat diet) were used in this study (Table 1). Gross energy of each diet (Table 1) was analyzed by oxygen bomb calorimetry (Model 6200, Oxygen Bomb Calorimeter, Parr Instrument, Moline, IL). Mice were shipped in two separate cohorts within 2 weeks. In each cohort, mice were randomized to 4 treatment groups (n = 11 per group for PAI-1^−/−^ mice fed the AIN93G or the high-fat diet, n = 14 per group for wild-type mice fed the AIN93G or the high-fat diet). Mice were maintained in a pathogen-free room on a 12∶12-hour light-dark cycle with a temperature of 22±1°C. Mice were weighed weekly, and they had free access to their diets and deionized water. Food intake was recorded for 3 weeks before cancer cell inoculation. All diets were powdered diets, and they were stored at −20°C until being provided to mice. Body composition was assessed in conscious, immobilized mice 1 week before cancer cell injection using quantitative magnetic resonance (Echo whole-body composition analyzer, Model 100, Echo Medical System, Houston, TX).

**Table 1:** Composition of experimental diets.

	AIN93G		High-Fat	
Ingredient	g	kcal	g	kcal
Corn starch	397.5	1590	33.5	134
Casein	200	800	200	800
Sucrose	100	400	100	400
Dextrin	132	528	200	800
Corn oil	70	630	201.5	1813.5
Cellulose	50	0	50	0
AIN93 mineral mix^a^	35	30	35	30
AIN93 vitamin mix^a^	10	39	10	39
L-cystine	3	12	3	12
Choline bitartrate	2.5	0	2.5	0
*tert*-butylhydroquinone	0.014	0	0.014	0
Total	1000	4029	835.5	4029
Calculated compositionfat, % kCal	16		45	
Analyzed composition				
gross energy, kCal/g^b^	4.37±0.01		5.27±0.05	

a Reference 21.

b n = 3 for each diet.

### Lewis lung carcinoma cells

Lewis lung carcinoma cell line, a variant that metastasizes specifically to lungs [22], was obtained from Dr. Pnina Brodt, McGill University, Montreal, Quebec, Canada. The cells were cultured with RPMI-1640 medium containing 10% heat-inactivated fetal bovine serum and maintained in a humidified atmosphere of 5% CO_2_ in air at 37°C. Cells used for animal studies were *in vivo*-selected once [19]. The cells were monitored for phenotype (by microscopic examination of cell morphology), proliferation properties (by growth curve analysis) and metastatic capability (by injecting cells subcutaneously into mice and examining metastatic formation in lungs). Cells were free of mycoplasma based on Hoechst DNA staining and direct culture tests performed by American Type Cell Collection (Manassas, VA). These assessments showed that cell identity and metastatic behavior were similar to those of original stocks from the institution providing the cell line.

### Experimental design

Mice (n = 22 per group for PAI-1^−/−^ mice, n = 28 per group for wild-type mice) were fed their respective AIN93G or high-fat diet for 7 weeks before they were subcutaneously injected with 2.5×10^5^ viable LLC cells per mouse into the lower dorsal region. The resulting primary tumor was removed surgically 12 days later when it was approximately 1 cm in diameter. Following removal, the mice were maintained on their respective diets for an additional 10 days. An additional 13 wild-type mice were fed the AIN93G diet but not injected with LLC cells. At the end of the study, plasma was collected from these mice to use as a control in comparison with AIN93G-fed LLC-bearing wild-type mice to assess the effect of metastasis on changes in plasma concentrations of adipokines, angiogenic factors and related biomarkers. Mice were terminated by intraperitoneal injection of a mixture of ketamine and xylazine. Lungs were removed and fixed with Bouin’s solution. The number of pulmonary metastases was counted [23] and the cross-sectional area and the volume of each metastasis were analyzed [24] using an ImagePro-Plus software- (Media Cybernetics, Silver Spring, MD) and a camera-equipped stereomicroscope. Tumor cross-sectional area was defined as the surface area of each lung metastasis. Tumor volume was estimated by assuming that tumors were spherical and using their average diameter [24]. The average diameter was the average measured at two degree intervals joining two outline points and passing through the centroid. Epididymal adipose tissue and plasma were collected and stored at −80°C for further analyses.

### Quantification of adipokines, angiogenic factors and related biomarkers

Concentrations of PAI-1 in epididymal adipose tissue, plasma and 24-hours-old conditioned medium from LLC cells were quantified using a sandwich enzyme-linked immunosorbent assay kit (Molecular Innovation, Novi, MI). An extract of adipose tissue was obtained by the protocol of Ortega et al [25]. Concentrations of PAI-1 in adipose extract and conditioned medium were normalized to protein content. Total protein concentration in each adipose extract and LLC cell lysate was estimated by BCA protein assay (Thermo Scientific, Waltham, MA). Plasma concentrations of uPA (Cell Sciences, Canton, MA), monocyte chemotactic protein-1 (MCP-1), tumor necrosis factor-α (TNF-α), leptin, vascular endothelial growth factor (VEGF), tissue inhibitor of metalloproteinase-1 (TIMP-1), insulin (all were from R&D Systems, Minneapolis, MN) and glucose (Cayman Chemical, Ann Arbor, MI) were quantified using sandwich enzyme-linked immunosorbent assay kits following manufacturers’ protocols. Samples were read within the linear range of the assay, and the accuracy of the analysis was confirmed by the controls provided in each kit.

### Statistical Analyses

The effects of diet (AIN93G or high-fat), genotype (PAI-1^−/−^ or wild-type) and their interaction were tested using two-way analysis of variance (ANOVA). If a significant interaction between diet and genotype occured, Tukey contrasts were performed to compare the 4 treatment groups. To examine the effect of metastasis on changes of plasma concentrations of adipokines, angiogenic factors and related biomarkers, *a priori* contrasts were used to test for differences in wild-type mice fed the AIN93G diet with or without LLC. A mixed model ANOVA with mouse as the random blocking factor and with diet, genotype and their interaction as fixed effects was used to test for differences in cross-sectional area and volume of metastases among the groups. All data are presented as means ± standard error of the mean (SEM). Differences with a *p*-value of 0.05 or less were considered statistically significant. All analyses were performed using SAS software (version 9.3, SAS Institute, Cary, NC).

## Results

**Figure 1:**
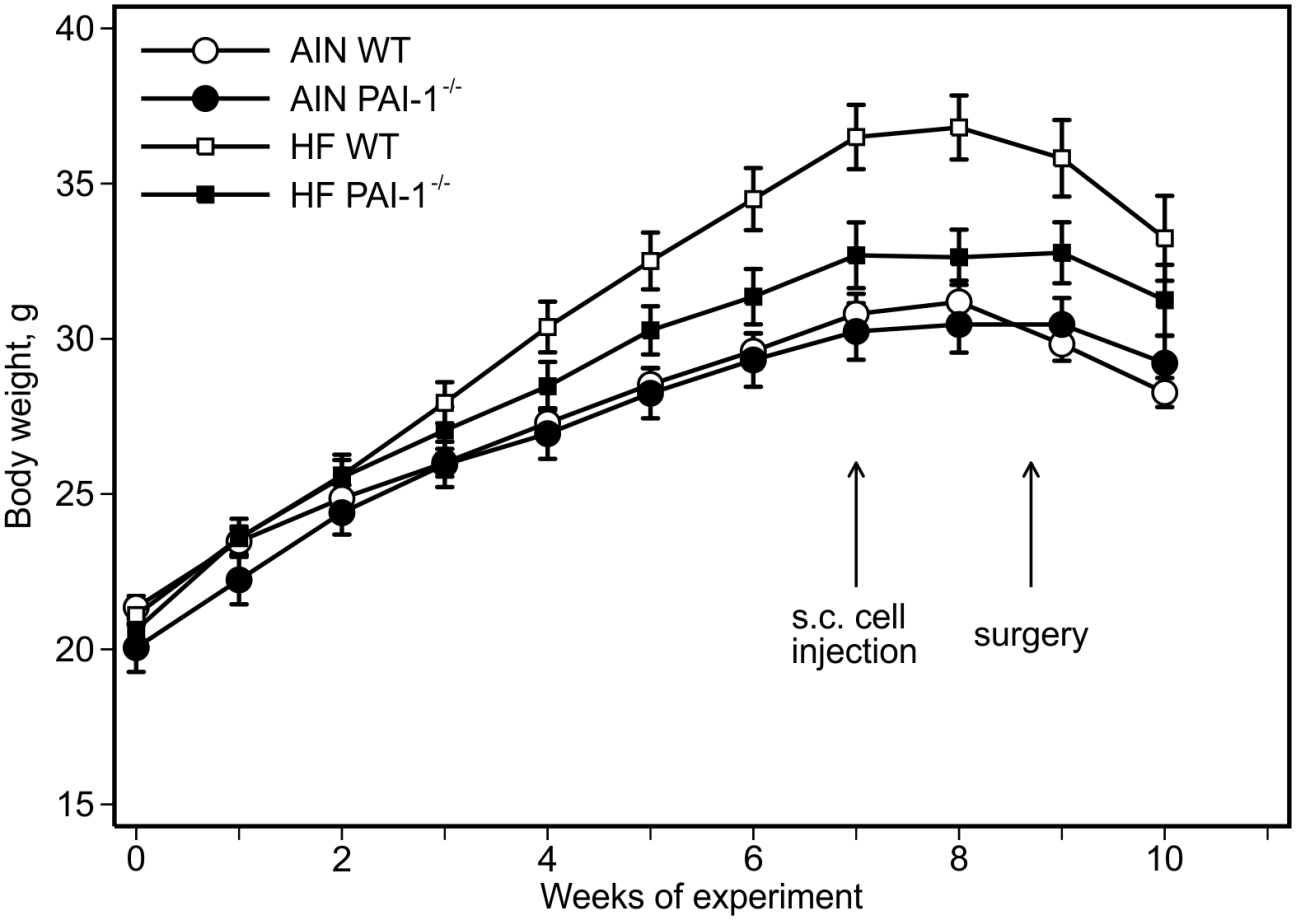
Body weight. Two-way ANOVA and Tukey contrasts were performed to test for differences among the groups. The high-fat diet, compared to the AIN93G diet, increased body weights; the difference was significant starting 4 weeks after the initiation of experimental feeding (*p*<0.01), and the significant increase continued throughout the experiment. Compared to wild-type mice, PAI-1 deficiency lowered body weights, and the difference was significant starting at week 6 of the experiment (*p*≤0.05). Values are means ± SEM (n = 11 per group for PAI-1^−/−^ mice, n = 14 per group for wild-type mice; second cohort). AIN WT: AIN93G-fed wild-type mice, AIN PAI-1^−/−^: AIN93G-fed PAI-1^−/−^ mice, HF WT: high-fat fed wild-type mice, HF PAI-1^−/−^: high-fat fed PAI-1^−/−^ mice.

Consumption of the high-fat diet increased body weights compared to the AIN93G diet. The difference was statistically significant 4 weeks after the initiation of experimental feeding (high-fat vs. AIN93G: *p*<0.01), and the significant increase continued throughout the experiment (Fig. 1). Starting at week 6, PAI-1^−/−^ mice weighed significantly less than wild-type mice (PAI-1^−/−^ vs. wild-type: *p*<0.05, Fig. 1), a difference more marked in the high-fat fed mice. Compared to the AIN93G diet, high-fat diet consumption increased body fat mass by 43% (high-fat vs. AIN93G: *p*<0.01) with a concomitant reduction in body lean mass by 9% (*p*<0.01) (Table 2). Compared to wild-type mice, PAI-1^−/−^ mice had a 15% reduction in fat mass (PAI-1^−/−^ vs. wild-type: *p*<0.01) and a 5% increase in lean mass (*p*<0.01) (Table 2). Neither the high-fat diet nor PAI-1 deficiency affected absolute lean mass weight (Table 2). Consumption of the high-fat diet increased caloric intake by 8% (high-fat vs. AIN93G: *p*<0.05) and PAI-1 deficiency lowered caloric intake by 9% (PAI-1^−/−^ vs. wild-type: *p*<0.05) compared to their respective controls (Table 2).

**Table 2:** Body fat mass ratio, lean mass ratio, absolute lean mass weight and caloric intake of mice^1^.

	Treatment				*p* value		
	AIN WT	AIN PAI-1^−/−^	HF WT	HF PAI-1^−/−^	Diet	Gene	Diet × Gene
Fat mass ratio, %	19.0±0.8	16.8±1.3	27.9±1.5	23.1±1.3	<0.01	<0.01	0.29
Lean mass ratio, %	72.0±0.8	73.9±1.1	64.1±1.4	68.3±1.1	<0.01	<0.01	0.32
Lean mass weight, g	21.1±0.3	21.9±0.4	21.8±0.3	22.2±0.4	0.12	0.07	0.45
Caloric intake, kCal/day	16.1±0.3	14.6±0.5	17.3±0.6	15.9±0.7	<0.05	<0.05	0.92

1 Two-way ANOVA was performed to test for differences among the groups. Values are means ± SEM (n = 22 per group for PAI-1^−/−^ mice, n = 28 per group for wild-type mice or n = 6 per group for caloric intake). AIN WT: AIN93G-fed wild-type mice, AIN PAI-1^−/−^: AIN93G-fed PAI-1^−/−^ mice, HF WT: high-fat fed wild-type mice, HF PAI-1^−/−^: high-fat fed PAI-1^−/−^ mice.

**Figure 2:**
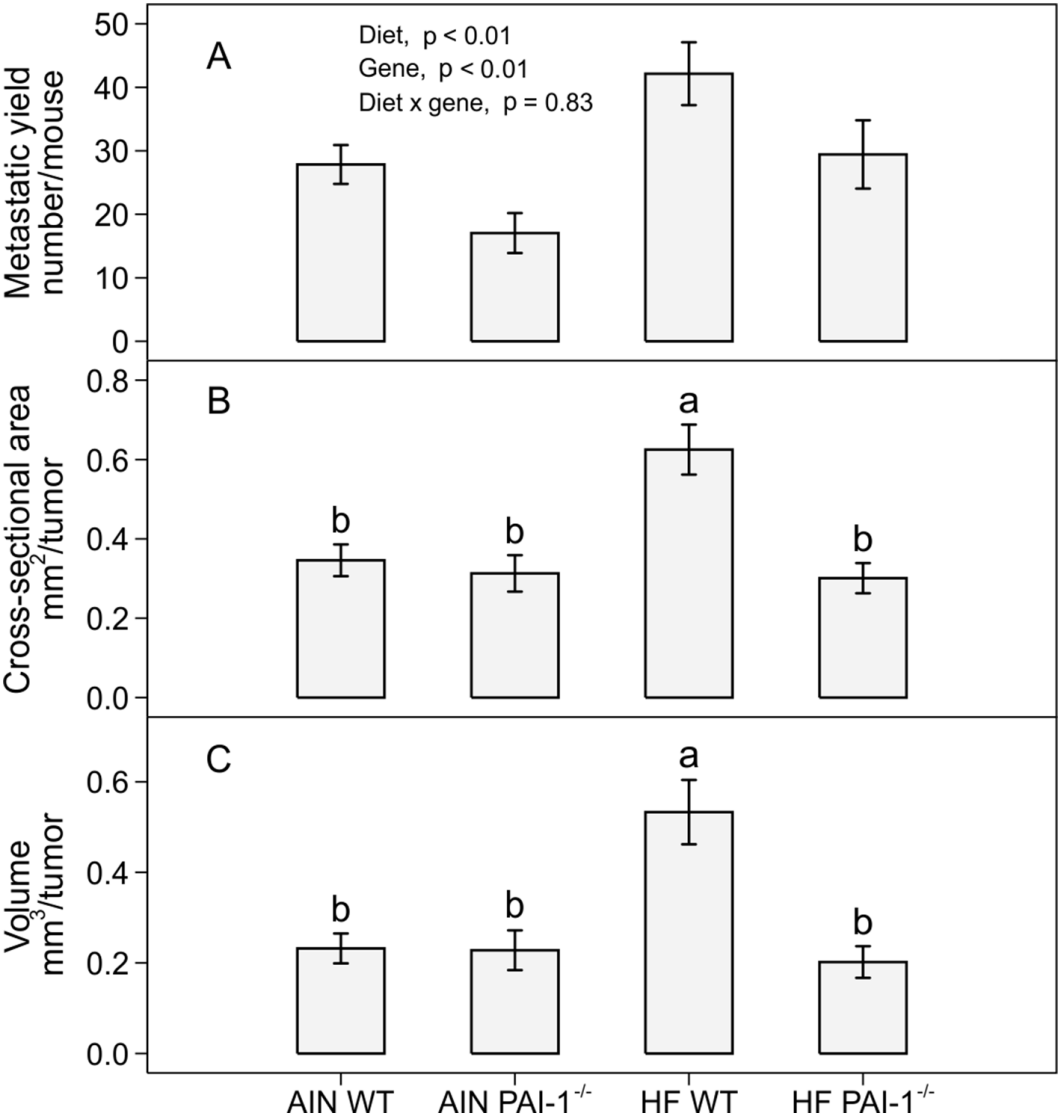
The number (*a*), cross-sectional area (*b*) and volume (*c*) of pulmonary metastases in PAI-1^−/−^ and wild-type mice fed the AIN93G or the high-fat diet. Two-way ANOVA and Tukey contrasts were performed to test for differences among the groups. Values (means ± SEM) with different superscripts are significantly different at *p*≤0.05 (n = 22 per group for PAI-1^−/−^ mice, n = 28 per group for wild-type mice). AIN WT: AIN93G-fed wild-type mice, AIN PAI-1^−/−^: AIN93G-fed PAI-1^−/−^ mice, HF WT: high-fat fed wild-type mice, HF PAI-1^−/−^: high-fat fed PAI-1^−/−^ mice.

Subcutaneous injection of LLC cells resulted in primary tumor and pulmonary metastasis. Compared to the AIN93G diet, high-fat diet consumption increased the number of lung metastases by 60% (high-fat vs. AIN93G: *p*<0.01; Fig. 2a). Mice deficient in PAI-1 had 35% less metastases than wild-type mice (PAI-1^−/−^ vs. wild-type: *p*<0.01; Fig. 2a). The high-fat diet increased tumor cross-sectional area by 82% (*p*<0.05, Fig. 2b) and tumor volume by 130% (*p*<0.05, Fig. 2c) in wild-type mice compared to AIN93G-fed controls. Deficiency in PAI-1 reduced tumor cross-sectional area by 52% (*p*<0.05, Fig. 2b) and tumor volume by 61% (*p*<0.05, Fig. 2c) in high-fat fed mice, but the deficiency did not affect either cross-sectional area or volume in mice fed the AIN93G diet (Fig. 2b, 2c), compared to their respective wild-type controls. Necropsy at the end of the study found no metastatic growth in other organs.

There was no significant difference in adipose PAI-1 between LLC-bearing and non-tumor-bearing mice fed the AIN93G diet (Table 3). Compared to the AIN93G diet, the high-fat diet increased adipose PAI-1 by 147% (*p*<0.05, Table 3). Lewis lung carcinoma increased plasma PAI-1 by 407% in wild-type mice compared to non-tumor-bearing controls (*p*<0.01, Table 3). The high-fat diet further increased plasma PAI-1 by 102% compared to the AIN93G diet (*p*<0.05, Table 3). Plasminogen activator inhibitor-1 in adipose tissue and plasma was not detectable in PAI-1^−/−^ mice regardless of the diet (Table 3). The conditioned medium from LLC cells contained 32.7±7.7 ng PAI-1/mg protein (n = 6).

**Table 3:** Adipose concentration of PAI-1 and plasma concentrations of adipokines (PAI-1, MCP-1, TNF-α, leptin) and uPA^1^.

	Treatment					*p* value		
	Control	AIN WT	AIN PAI-1^−/−^	HF WT	HF PAI-1^−/−^	Diet	Gene	D × G
PAI-1,^2 ^ng/mg protein	0.22±0.03	0.17±0.04^b^	N.D.	0.42±0.07^a^	N.D.	<0.01	<0.01	<0.01
PAI-1, ng/mL	0.44±0.10*	2.23±0.30^b^	N.D.	4.50±0.48^a^	N.D.	<0.01	<0.01	<0.01
uPA, ng/mL	0.82±0.06*	1.76±0.20	1.51±0.23	3.77±0.35	3.15±0.33	<0.01	0.14	0.51
MCP-1, pg/mL	7.32±1.97*	39.77±4.85^b^	43.21±2.27^b^	87.77±3.65^a^	114.88±8.37^a^	<0.01	<0.01	<0.05
TNF-α, pg/mL	1.93±0.64*	9.60±0.30	10.53±0.32	16.51±0.39	17.49±0.47	<0.01	<0.05	0.94
Leptin, ng/mL	4.53±0.42	4.57±0.35	6.48±0.60	10.18±0.75	12.53±1.14	<0.01	0.01	0.77

1 Two-way ANOVA and Tukey contrasts were performed to compare differences among the groups of LLC-bearing mice; *a priori* contrasts were performed to compare differences in AIN93G-fed wild-type mice with or without LLC (control vs. AIN WT). Values (means ± SEM) in a row with different superscripts are significantly different at *p*≤0.05 for LLC-bearing groups (n = 10 per group).

*p<0.01 compared to AIN WT. Control: AIN93G-fed non-tumor-bearing wild-type mice, AIN WT: AIN93G-fed wild-type mice, AIN PAI-1^−/−^: AIN93G-fed PAI-1^−/−^ mice, HF WT: high-fat fed wild-type mice, HF PAI-1^−/−^: high-fat fed PAI-1^−/−^ mice, D × G: diet × gene interaction, N.D.: not detectable.

2 Adipose PAI-1.

Plasma levels of uPA were increased in tumor-bearing mice by 115% compared to non-tumor-bearing controls (*p*<0.01, Table 3). Compared to the AIN93G diet, the high-fat diet increased plasma uPA by 112% (high-fat vs. AIN93G: *p*<0.01, Table 3). There was no significant difference in plasma uPA between PAI-1^−/−^ and wild-type mice (Table 3).

Compared to non-tumor-bearing controls, mice with LLC had a 443% increase in plasma MCP-1 (*p*<0.01) and a 397% increase in TNF-α (*p*<0.01) (Table 3). The high-fat diet, compared to the AIN93G diet, increased MCP-1 by 144% (high-fat vs. AIN93G: *p*<0.01) and TNF-α by 69% (high-fat vs. AIN93G: *p*<0.01) (Table 3). Compared to wild-type mice, PAI-1^−/−^ mice had a 24% increase in plasma MCP-1 (PAI-1^−/−^ vs. wildtype: *p*<0.01) and 7% increase in TNF-α (PAI-1^−/−^ vs. wildtype: *p*<0.05) (Table 3). Lewis lung carcinoma did not affect plasma levels of leptin (Table 3). The high-fat diet, compared to the AIN93G diet, increased plasma leptin by 106% (high-fat vs. AIN93G: *p*<0.01, Table 3). Plasminogen activator inhibitor-1 deficiency increased leptin by 29% (PAI-1^−/−^ vs. wild-type: *p* = 0.01, Table 3).

Mice with LLC exhibited an 11% increase in plasma concentrations of VEGF (*p*<0.01) and a 47% increase in TIMP-1 (*p*<0.01) compared to non-tumor-bearing controls (Table 4). The high-fat, compared to the AIN93G diet, increased plasma VEGF by 12% (high-fat vs. AIN93G: *p* = 0.01) and TIMP-1 by 71% (high-fat vs. AIN93G: *p*<0.01) (Table 4). Deficiency in PAI-1 increased plasma VEGF by 11% (PAI-1^−/−^ vs. wild-type: *p*<0.05) and TIMP-1 by 27% (PAI-1^−/−^ vs. wild-type: *p*<0.01) (Table 4).

**Table 4:** Plasma concentrations of angiogenic factors (VEGF, TIMP-1), insulin and glucose^1^.

	Treatment					*p* value		
	Control	AIN WT	AIN PAI-1^−/−^	HF WT	HF PAI-1^−/−^	Diet	Gene	D × G
VEGF, pg/mL	69.41±1.23*	77.1±2.86	84.34±5.98	85.00±1.76	96.16±2.97	0.01	<0.05	0.60
TIMP-1, ng/mL	0.81±0.04*	1.19±0.09	1.49±0.08	2.01±0.10	2.57±0.11	<0.01	<0.01	0.18
Insulin, ng/mL	0.32±0.01	0.32±0.01^b^	0.31±0.01^b^	0.59±0.01^a^	0.74±0.05^a^	<0.01	0.01	<0.01
Glucose, mg/mL	0.84±0.04	0.82±0.04	0.87±0.03	1.08±0.03	1.00±0.03	<0.01	0.64	0.07

1 Two-way ANOVA and Tukey contrasts were performed to compare differences among the groups of LLC-bearing mice; *a priori* contrasts were performed to compare differences in AIN93G-fed wild-type mice with or without LLC (control vs. AIN WT). Values (means ± SEM) in a row with different superscripts are significantly different at *p*≤0.05 for LLC-bearing groups (n = 10 per group). *p<0.01 compared to AIN WT. Control: AIN93G-fed non-tumor-bearing wild-type mice, AIN WT: AIN93G-fed wild-type mice, AIN PAI-1^−/−^: AIN93G-fed PAI-1^−/−^ mice, HF WT: high-fat fed wild-type mice, HF PAI-1^−/−^: high-fat fed PAI-1^−/−^ mice, D × G: diet × gene interaction.

There were no differences in plasma concentrations of insulin and glucose between LLC-bearing and non-tumor-bearing mice (Table 4). The high-fat diet, compared to the AIN93G diet, increased plasma insulin by 111% (high-fat vs. AIN93G: *p*<0.01) and glucose by 23% (high-fat vs. AIN93G: *p*<0.01) (Table 4). Deficiency in PAI-1 increased plasma insulin by 15% (PAI-1^−/−^ vs. wild-type: *p* = 0.01, Table 4). There was no significant difference in plasma glucose between PAI-1^−/−^ and wild-type mice (Table 4).

## Discussion

The present study demonstrates that PAI-1 participates in spontaneous metastasis of LLC and that PAI-1 deficiency reduces metastasis enhanced by the high-fat diet.

Malignant spread is an important and critical step of metastatic development. The finding of a significant reduction in pulmonary metastases in PAI-1^−/−^ mice indicates that host-produced PAI-1 may play an active role in metastasis. This is supported by previous reports that the absence of host PAI-1 prevents invasion of transplanted malignant keratinocytes *in vivo* [6]. Plasma concentrations of uPA in PAI-1^−/−^ mice were similar to those of their wild-type counterparts, indicating that the action of PAI-1 on cancer spread may be independent on uPA. However, there is evidence suggesting that both PAI-1 and uPA are necessary for maximal metastatic progression. Lung cancer cells expressing only uPA and its receptor display no invasive capability [26].

When fed the high-fat diet, PAI-1^−/−^ mice had a significant smaller metastasis size compared to the wild-type mice. This finding indicates that PAI-1 expression is needed for high-fat feeding enhancement of growth of metastases.

Adipose PAI-1 was depleted in PAI-1^−/−^ mice, regardless of the diet. This finding indicates that it is the PAI-1 derived from adipose tissues that participates in LLC progression and contributes to enhanced malignant spread and growth by the high-fat diet. Although LLC produces PAI-1, the present results suggest that the amount of PAI-1 contributed by LLC in this model is insufficient to overcome the host deficiency of this adipokine. It indicates the importance of exogenous PAI-1 in metastasis of PAI-1 dependent malignancies.

The present study demonstrates that plasma concentrations of inflammatory cytokines (MCP-1, TNF-α, leptin), angiogenic factors (VEGF, TIMP-1) and insulin are elevated in PAI-1^−/−^ mice. These elevations apparently compromise a compensation for the absence of PAI-1. Monocyte chemotactic protein-1 [27] and TNF-α [28], [29] are tumorigenic and angiogenic. Leptin is known to be angiogenic [30] and to modulate tumor growth by increasing VEGF expression [31]. Vascular endothelial growth factor [32] and TIMP-1 [33] contribute to metastatic development. Hyperinsulinemia enhances growth and progression of mammary tumors [34], [35]. Thus, observed elevations of these factors suggest that they may contribute to the less aggressive development and growth of LLC in the absence of PAI-1 in PAI-1^−/−^ mice.

Plasminogen activator inhibitor-1 together with uPA has been recommended as prognostic biomarkers in breast cancer [36]. RNA aptamers specific to PAI-1 were generated for preclinical testing for potential clinical applications [37]. PAI-039, a small molecule inhibitor of PAI-1 that was investigated clinically for treatments of acute and arterial thrombosis [38], has been tested for its efficacy in preclinical tumor angiogenesis mouse models [39]. The present results suggest that treatments targeting PAI-1 may be counteracted by the compensatory overproduction of inflammatory cytokines and angiogenic factors. This warrants further investigation, particularly in those studies aimed at long-term PAI-1 inhibition.

That PAI-1 was not detectable in plasma from LLC-bearing PAI-1^−/−^ mice suggests that LLC-produced PAI-1 is not responsible for the significant elevated plasma PAI-1 in AIN93G-fed wild-type mice with LLC. Plasminogen activator inhibitor-1 is an acute phase reactant [40]. The concentration of PAI-1 in mouse liver is relatively low [41]; however, PAI-1 gene expression in the liver is up-regulated markedly by endotoxin and inflammatory mediators [41], [42]. Because cancer has an inflammatory component, the presence of LLC in wild-type mice may be up-regulating PAI-1 production from liver and other organs, resulting in significantly higher plasma PAI-1 than in the non-tumor-bearing wild-type mice.

Reductions in caloric intake and body fat mass in PAI-1^−/−^ mice fed the high-fat diet may have contributed to the inhibition of malignant progression, particularly metastatic growth, in those mice. Dietary energy restriction reduces primary tumorigenesis and metastasis [43]. Caloric restriction, which lowers adiposity and production of inflammatory cytokines, may be a useful adjuvant in the prevention and treatment of obesity-related cancer. The present results suggest that better outcomes in studies aimed at PAI-1 inhibition may come from subjects with healthy body weights, whereas overweight/obesity or excessive caloric intake may be additive to PAI-1 deficiency-related compensatory increases in inflammatory cytokines and angiogenic factors.

Significant reduction in body fat mass and increases in plasma concentrations of leptin and insulin in PAI-1^−/−^ mice were not expected considering the existing understanding that blood levels of leptin and insulin are positively associated with adiposity [44], [45]. However, it has been reported that plasma insulin was increased in PAI-1^−/−^ mice compared to wild-type mice, even though there was no difference in gonadal fat pad weight between the groups [46]. Considering the significant increases in adipokines and angiogenic factors in PAI-1^−/−^ mice in the present study, the increase in leptin, as well as insulin, is likely through an overproduction compensatory mechanism in the absence of PAI-1, which may be independent of body fat mass or body weight.

That LLC-associated increases in plasma concentrations of inflammatory cytokines PAI-1, MCP-1, TNF-α, protease uPA and angiogenic factors VEGF, TIMP-1 indicate the aggressiveness of this carcinoma model. High expressions of PAI-1 [2], [47], MCP-1 [48], [49], TNF-α [50], uPA [2], VEGF [51] and TIMP-1 [52] have been associated with regional or distant metastasis and poor prognosis in cancer patients. Elevations of these cytokines [3], [53], [54], uPA [55], [56] and angiogenic markers [19], [33] have been reported in animal models of various malignancies. The present results are consistent with the existing knowledge and confirm the usefulness of the LLC model to study spontaneous metastasis.

The present study used corn oil as the source of dietary fat, which is comprised of 57% linoleic acid [57] and provided 25% of total energy in the high-fat diet. This diet was low in α-linolenic acid (0.5% of dietary energy), which comprises 1% of fatty acids in corn oil [57]. It is possible that this low α-linolenic acid/linoleic acid ratio [58] may have contributed to the effects observed for the high-fat diet because of its low content of α-linolenic acid, which has been suggested to be anticarcinogenic [59], [60]. This possibility warrants further investigation.

In summary, results from the present study indicate that PAI-1 contributes to the metastasis of LLC and that PAI-1 deficiency ameliorates metastasis enhanced by the high-fat diet. These findings are consistent with others showing that PAI-1 participates in cancer progression [3], [4]. Furthermore, the present study demonstrates that host-produced PAI-1 including that from adipose tissues was a significant determinant of malignant progression. Adipose tissue is a modifiable source of PAI-1, particularly in obesity. Weight loss through caloric restriction alone or in combination with exercise reduces blood levels of PAI-1 [61], [62]; thus, these practices can be useful adjuvants to achieving the best outcomes of chemoprevention. The compensatory increases in production of inflammatory cytokines, angiogenic factors and insulin in PAI-1^−/−^ mice suggest that these compensatory responses may counteract treatments aimed at PAI-1 inhibition.
