# Correction: Effects of a High-Fat Diet on Spontaneous Metastasis of Lewis Lung Carcinoma in Plasminogen Activator Inhibitor-1 Deficient and Wild-Type Mice

**DOI:** 10.1371/journal.pone.0118115

**Published:** 2015-03-30

**Authors:** The PLOS ONE Staff

There is an error in columns 2 and 3. The values 16 and 45 should align with the lower half of the cell. Please view the correct Table 1 here.

**Table 1:** Composition of experimental diets.

	AIN93G		High-Fat	
Ingredient	g	kcal	g	kcal
Corn starch	397.5	1590	33.5	134
Casein	200	800	200	800
Sucrose	100	400	100	400
Dextrin	132	528	200	800
Corn oil	70	630	201.5	1813.5
Cellulose	50	0	50	0
AIN93 mineral mix ^a^	35	30	35	30
AIN93 vitamin mix ^a^	10	39	10	39
L-cystine	3	12	3	12
Choline bitartrate	2.5	0	2.5	0
*tert*-butylhydroquinone	0.014	0	0.014	0
Total	1000	4029	835.5	4029
Calculated composition				
fat, % kCal	16		45	
Analyzed composition				
gross energy, kCal/g ^b^	4.37 ± 0.01		5.27 ± 0.05	

^a^ Reference 21.

^b^ n = 3 for each diet.
